# Kaposi’s sarcoma-associated herpesvirus T cell responses in HIV seronegative individuals from rural Uganda

**DOI:** 10.1038/s41467-021-27623-8

**Published:** 2021-12-16

**Authors:** Angela Nalwoga, Romin Roshan, Kyle Moore, Vickie Marshall, Wendell Miley, Nazzarena Labo, Marjorie Nakibuule, Stephen Cose, Rosemary Rochford, Robert Newton, Denise Whitby

**Affiliations:** 1 MRC/UVRI and LSHTM Uganda Research Unit, Entebbe, Uganda; 2 Department of Immunology and Microbiology, University of Colorado, Anschutz Medical Campus, Aurora, CO USA; 3 Viral Oncology Section, AIDS and Cancer Virus Program, Leidos Biomedical Research, Inc., Frederick National Laboratory for Cancer Research, Frederick, MD USA; 4 London School of Hygiene & Tropical Medicine, London, UK; 5 University of York, York, UK

**Keywords:** Tumour virus infections, Cellular immunity, T cells, Herpes virus

## Abstract

T cell responses to Kaposi’s sarcoma-associated herpesvirus (KSHV) are likely essential in the control of KSHV infection and protection from associated disease, but remain poorly characterised. KSHV prevalence in rural Uganda is high at >90%. Here we investigate IFN- γ T cell responses to the KSHV proteome in HIV-negative individuals from a rural Ugandan population. We use an ex-vivo IFN- γ ELISpot assay with overlapping peptide pools spanning 83 KSHV open reading frames (ORF) on peripheral blood mononuclear cells (PBMC) from 116 individuals. KSHV-specific T cell IFN- γ responses are of low intensity and heterogeneous, with no evidence of immune dominance; by contrast, IFN- γ responses to Epstein–Barr virus, Cytomegalovirus and influenza peptides are frequent and intense. Individuals with KSHV DNA in PBMC have higher IFN- γ responses to ORF73 (*p* = 0.02) and lower responses to K8.1 (*p* = 0.004) when compared with those without KSHV DNA. In summary, we demonstrate low intensity, heterogeneous T cell responses to KSHV in immune-competent individuals.

## Abstract (web summary)

The T cell response to Kaposi sarcoma-associated herpesvirus is critical to controlling infection and immunopathology but has been poorly explored in immunocompetent patients. Here the authors characterise the T cell response to the Kaposi sarcoma-associated herpesvirus proteome in immunocompetent patients from rural Uganda.

## Introduction

The adaptive response to Kaposi sarcoma-associated herpesvirus (KSHV) is important in protecting from KSHV-associated malignancies, i.e., Kaposi’s sarcoma (KS), multicentric Castleman’s disease and primary effusion lymphoma^1,2^. This is shown by the substantial increase in the risk of developing KS- and other KSHV-associated malignancies in immunocompromised individuals^3,4^. A higher frequency of KSHV-specific T cells has been reported in KS patients who achieve complete remission compared to cases with unresolved KS^5–7^. In comparison to KS patients, more frequent and higher KSHV-specific T cell responses have been reported in asymptomatic individuals^8^. Treatment with highly active antiretroviral therapy of AIDS-KS patients has been associated with an improved KSHV-specific T cell response, correlating with KS regression^9,10^. Furthermore, the pivotal role of T cells in controlling other herpesviruses, including Epstein–Barr virus (EBV), CMV and HSV, has been well investigated^11,12^, although similar evidence (specific requirements and immunodominant epitopes) for KSHV is less well understood. Understanding T cell responses to KSHV is important for the development of KSHV vaccines and immunotherapy for KS.

During the adaptive immune response to viral infections, CD8+ T cells play a vital role in killing virally-infected cells and producing effector cytokines, while CD4+ T cells produce cytokines to enhance CD8+ T cells cytotoxic killing of infected cells. Several studies have reported a predominant role of cytotoxic CD8+ T cells in the control of classic, iatrogenic and AIDS-related KS^1,2,13,14^. However, these studies have focused on HLA-A2 epitopes, a frequent haplotype in Caucasians^15^, and were performed in mostly, non-KS endemic areas, with only two studies reporting cellular responses to KSHV in sub-Saharan Africa and only then in a limited number of study participants^16,17^.

The ~165 kB double-stranded DNA KSHV genome encodes about 90 proteins^18^ with a very high number of potential T cell epitopes in each protein. Previous studies have identified epitopes in individual participants, but these have not been confirmed in other studies. We developed a systematic approach using overlapping peptides to the entire KSHV proteome to investigate KSHV cellular immunity in US healthy donors and patients with KSHV-related disease^19,20^. We made peptide pools corresponding to each KSHV open reading frame (ORF) and measured responses by IFN-γ ELISpot. We showed that participants responded typically to between one and five peptide pools but, that there was no overlap between participants in the peptide pools recognised. Responses were heterogeneous and generally low compared to responses to EBV and CMV and suggested a lack of immunodominance.

Detection of KSHV DNA as well as its quantity in peripheral blood mononuclear cells (PBMC) has been associated with KS disease progression^21–24^ and increased HIV viremia in HIV-infected individuals^25^. In individuals without KS- or any other KSHV-associated disease, we have previously shown that KSHV DNA in PBMC was associated with increased KSHV antibody levels to lytic KSHV protein K8.1 and to malaria parasitaemia^26^. An association between increased lytic KSHV antibody levels and KS disease risk has also been reported^27^. Similarly in KS patients, correlations between KSHV in PBMC with increasing KSHV lytic antibody titres have been shown^25^. In combination, these data suggest that KSHV in PBMC corresponds to viral reactivation and disease progression. Therefore we hypothesise that loss of KSHV T cell control leads to increased KSHV DNA in PBMC.

In the current study, we use our systematic approach to investigate T cell responses to nearly the entire KSHV proteome in 116 KSHV+HIV-negative individuals with a wide age range from a rural cohort in Uganda, where both KSHV and KS are endemic. In addition, we compare T cell responses among KSHV seropositive individuals with and without KSHV DNA in PBMC.

## Results

### Study population

A total of 116 KSHV seropositive individuals aged 6–87 years are included in the analysis. Of these, 13% (15/116) are 6–17 years old, 74% (86/116) are 18–50 years old and 13% (15/116) are 51–87 years of age. Overall, 51% (59/116) are male and 41% (47/116) have detectable KSHV DNA in PBMC (Table 1).

**Table 1:** Proportions of individuals responding with IFN-γ production and their overall characteristics.

All individuals analysed (*n* = 116)	
Age mean (range)	34 (6–87)
6–17 years	13% (15/116)
18–50 years	74% (86/116)
51–87 years	13% (15/116)
Sex, males	51% (59/116)
Proportion of individuals with KSHV DNA in PBMC	41% (47/116)
Proportion of individuals with a positive IFN-γ response to at least one KSHV ORF	77% (89/116)
Proportion of individuals with positive IFN-γ responses to one to two KSHV ORF	44% (51/116)
Proportion of individuals with positive IFN-γ responses to three or more (up to 37) KSHV ORF	33% (38/116)
Proportion of individuals with positive IFN-γ responses to CEF^a^	69% (80/116)
Proportion individuals with positive IFN-γ responses to EBV	76% (58/76)

KSHV DNA measured using real-time PCR, IFN-γ responses of PBMC were detected and quantified using an ex vivo ELISpot assay.

Source data are provided as a Source Data file.

*PBMC* peripheral blood mononuclear cell.

^a^CMV + EBV + flu (CEF) peptides cocktail.

### The pattern of IFN-γ responses to KSHV antigens compared to controls (EBV and CEF)

Most of these individuals respond to EBV (76%) and CEF (69%) antigens (Table 1). Eighty-nine individuals out of 116 have a positive IFN−γ response to at least one KSHV ORF (77%); 44% respond to one to two KSHV ORFs and 33% respond to three or more KSHV ORFs. One individual responds to 37 KSHV ORFs (Table 1).

**Fig. 1:**
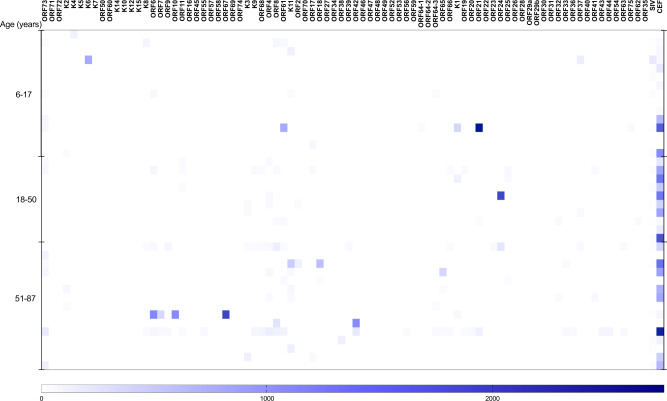
KSHV-specific IFN-γ responses of 40 Ugandans aged 6–87 years. Ex vivo ELISpot assay was used to determine IFN-γ responses to KSHV overlapping peptide pools. Spot forming cells (SFC) per million PBMCs were recorded for each reaction. The intensity of the purple colour correlates with the number of SFC per million PBMCs. The raw data used to draw this graph are shown in Supplementary Data 2. SIV Simian immunodeficiency virus, CEF CMV + EBV + flu cocktail, EBV Epstein–Barr virus. Heatmap drawn in GraphPad Prism version 8.0.1. Source data are provided as a Source Data file.

**Fig. 2:**
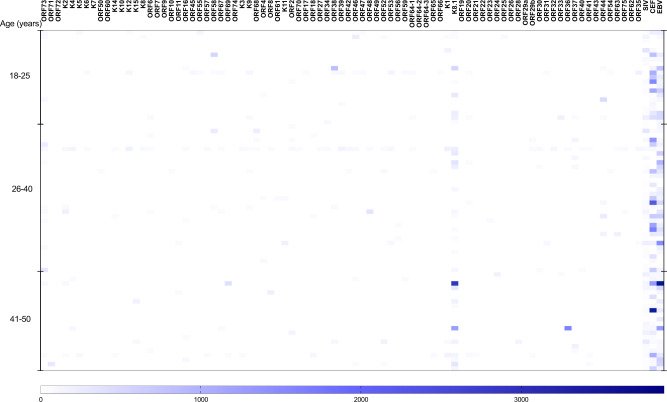
KSHV-specific IFN-γ responses of 76 Ugandans aged 18–50 years. Ex vivo ELISpot assay was used to determine IFN-γ responses to KSHV overlapping peptide pools. Spot forming cells (SFCs) per million PBMCs were recorded for each reaction. The intensity of the purple colour correlates with the number of SFCs per million PBMCs. The raw data used to draw this graph are shown in Supplementary Data 1. SIV Simian immunodeficiency virus, EBV Epstein–Barr virus, CEF CMV + EBV + flu cocktail. Heatmap drawn in GraphPad Prism version 8.0.1. Source data are provided as a Source Data file.

**Fig. 3:**
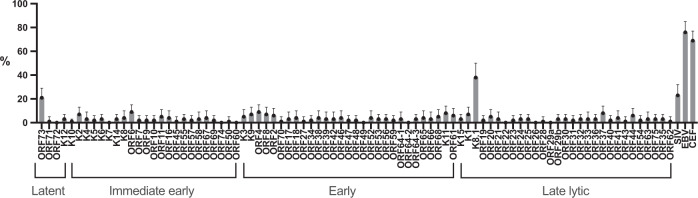
Proportion of individuals with IFN-γ responses to various KSHV ORFs and controls peptide pools. Dots represent the percentage of individuals reacting to the different KSHV antigens. Error bars represent 95% confidence intervals. *N* = 116 study participants. SIV Simian immunodeficiency virus, EBV Epstein–Barr virus, CEF CMV + EBV + flu cocktail. Graph drawn in GraphPad Prism version 8.0.1. Source data are provided as a Source Data file.

**Fig. 4:**
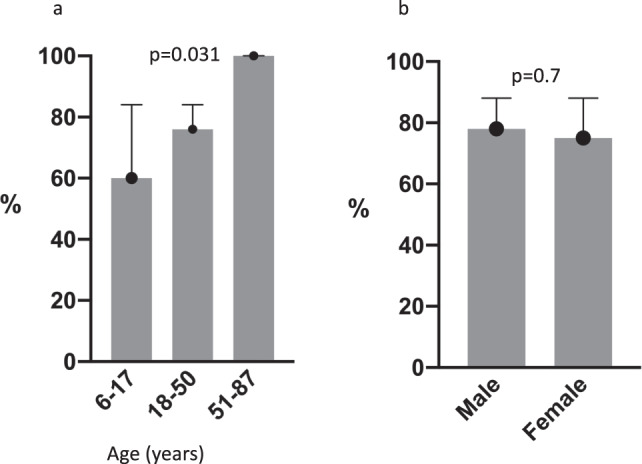
Proportion of individuals responding to atleast one KSHV ORF. . Individuals categorized by age group (**a**) and sex (**b**).

Next, we compare the magnitude of the IFN-γ responses. The magnitude of IFN-γ responses to CEF (Figs. 1 and 2) and EBV (Fig. 2) range from 40 spot forming cell (SFC)/million PBMC to 3713 and 3891–SFC/million PBMC, respectively (Supplementary Fig. 1). There is no consistent pattern of response to KSHV ORFs (Fig. 3), and when an IFN-γ response is observed to individual KSHV ORFs, the magnitude of the response is generally low (Figs. 1 and 2 and Supplementary Data 1 and 2). The raw data used to draw Fig. 1 are shown in Supplementary Data 1, while the raw data used to draw Fig. 2 are shown in Supplementary Data 2. Furthermore, there is no pattern observed either by age group or sex. However, we observe that the proportion of individuals responding to at least one KSHV ORF increased with age (Fig. 4a) and is similar in males and females (Fig. 4b). Furthermore, the number of reactive KSHV ORFs per individual increases with the increasing age of the study participant (Supplementary Fig. 2). Among KSHV peptide pools, K8.1 is the most commonly recognised ORF (38%) followed by ORF73 (21%) (Fig. 3). Surprisingly, 23% of individuals respond to a Simian immunodeficiency virus (SIV) peptide (Fig. 3).

### IFN-γ responses K8.1 and ORF73 by KSHV DNA detection in PBMC

**Fig. 5:**
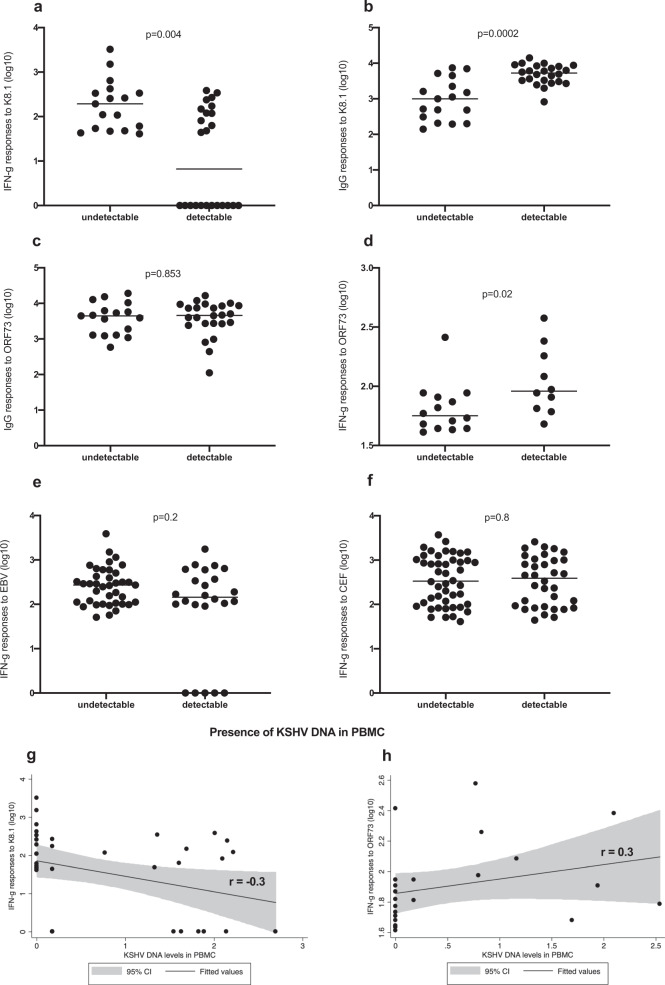
Immune responses (IFN-y) and IgG antibody) to K8.1 and ORF73 as well as IFN-y responses to EBV and CMV+EBV+flu cocktail-CEF in individuals with and without KSHV DNA in peripheral blood mononuclear cells (PBMC). **a**, **g** IFN-y responses to K8.1; **b** IgG antibody responses to K8.1; **c** IgG antibody responses to ORF73; **d**, **h** IFN-y responses to ORF73; **e** IFN-y responses to EBV; **f** IFN-y responses to CEF.

Since K8.1 and ORF73 are the most frequently recognised ORFs (45% of individuals react to either K8.1 or ORF73 and 11% react to both), we determine if IFN-γ responses to K8.1, ORF73 and positive controls (EBV and CEF) are related to the detection of KSHV DNA in PBMC. Individuals without KSHV DNA in PBMC have higher IFN-γ responses to K8.1 compared to those with KSHV DNA in PBMC (Fig. 5a, g). Furthermore, there is a trend towards a negative correlation between IgG antibody levels to K8.1 and IFN-γ responses to K8.1 (Supplementary Fig. 3). As previously reported^26^, anti-K8.1 IgG antibody levels are higher in those with detectable KSHV DNA in PBMC (Fig. 5b) while no difference is observed with anti-ORF73 antibody levels (Fig. 5c). Conversely, individuals without KSHV DNA in PBMC have lower IFN-γ responses to ORF73 compared to those without KSHV DNA in PBMC (Fig. 5d, h). There is no difference in IFN-γ responses to both EBV (Fig. 5e) and CEF (Fig. 5f) among individuals with and without KSHV DNA in PBMC.

### IFN-γ responses to individual peptides within the K8.1 peptide pool

Results from four samples tested for K8.1 individual peptide IFN-γ responses show diversity in the responses. The individuals’ GPC-69, GPC-313, GPC-371 and GPC-339 responded to 10, 7, 2 and 1 K8.1 peptides, respectively (Table 2). Three (GPC-69, GPC-371 and GPC-339) out of four individuals responded to peptide 44 (peptide sequence: YLCVPRCRRKKPYIV).

**Table 2:** Identification of responding peptides within the K8.1 peptide pool (44 peptides).

Study participant identification	Number of peptides (within K8.1 pool) recognised per study participant	Peptides recognised (spot count per million PBMC)
GPC-313	7	10 (64), 17 (56), 30 (112), 34 (56), 35 (80), 36 (92), 41 (104)
GPC-69	10	22 (87), 29 (73), 31 (133), 34 (93), 36 (327), 37 (180), 38 (67), 41 (247), 43 (320), 44 (333)
GPC-339	1	44 (93)
GPC-371	2	43 (200), 44 (1627)

Ex vivo IFN-γ ELISpot assay was used to identify responding peptides within the K8.1 peptide pool using a grid-type method^20^. Sequences of responding peptides: 10: VYQDWLGRMNCSYEN, 17: SEYPNVSVSVEDTSA, 22: SGSGEEERPVTSHVT, 29: SGSYSSGEPSRTTRI, 30: SGEPSRTTRIRVSPV, 31: RTTRIRVSPVAENGR, 34: NSGASNRVPFSATTT, 35: NRVPFSATTTTTRGR, 36: SATTTTTRGRDAHYN, 37: TTRGRDAHYNAEIRT, 38: DAHYNAEIRTHLYIL, 41: WAVGLLLGLVLILYL, 43: LILYLCVPRCRRKKP, 44: YLCVPRCRRKKPYIV.

Source data are provided as a Source Data file.

*PBMC* peripheral blood mononuclear cell, *GPC* General Population Cohort.

### Number of reactive antigens per individual in relation to KSHV DNA in PBMC

**Fig. 6:**
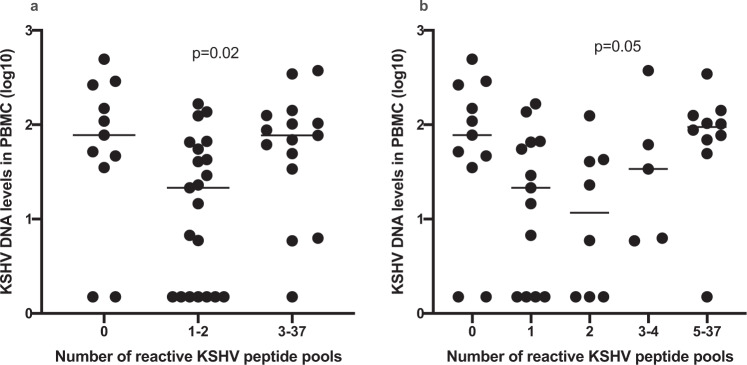
KSHV DNA levels in peripheral blood mononuclear cells (PBMC) by number of reactive peptide pools. (**a**) individuals grouped in 3 categories; (**b**) individuals grouped in 5 categories.

As mentioned above, 23%, 44% and 33% of individuals respond to no KSHV antigen, 1–2 antigens and 3–37 antigens, respectively. To explore whether there is a correlation between detectable levels of KSHV in PBMC, which could be indicative of a loss of T-cell control, we then compare the three groups (no response, response to 1–2 antigens or 3–37 antigens) focusing on those with KSHV DNA in PBMC. Individuals who respond to 1–2 KSHV antigens have the lowest levels of KSHV DNA in PBMC, while those without any IFN-γ response to KSHV and those with IFN-γ responses to three or more KSHV antigens have similar levels of KSHV DNA in PBMC (Fig. 6a). When we broke the groups into five smaller groups, we observe a decline in KSHV viral load from the non-responders to those responding to two peptide pools, then an increase in KSHV viral load thereafter (Fig. 6b). The low levels of KSHV viral load among those responding to 1–2 peptide pools and the higher levels of KSHV viral load among those responding to 3 or more peptide pools could be explained by the fact that very few individuals reacting to ORF73 were in the 1–2 peptide pool group.

## Discussion

Previous studies showed that individuals without KS disease have higher T cell responses compared to those with KS^8,28–30^ emphasising a key contribution of insufficient KSHV T cell responses to the risk of KS disease. Contrary to this, we have shown previously that US patients with KSHV-related disease responded to a greater number of ORFs compared to asymptomatic KSHV-infected healthy participants, but the magnitude of the responses was similar^20^. Others have also shown that individuals with HIV infection have low KSHV-specific T cell responses compared to HIV-negative individuals, likely due to the effect of HIV on CD4 T cells^31,32^. Furthermore, the reduction in HIV viral load and increase in CD4 count correlated with increased KSHV-specific T cell immunity^7^. In light of these mixed findings, studies investigating KSHV T cell responses in HIV-negative individuals were needed^33^. We, therefore, in the current study focus on HIV-negative individuals without any apparent form of KSHV-associated disease who are KSHV seropositive and from a KSHV/KS endemic area, in order to understand KSHV T cell responses in the absence of overt immune suppression. In this study, we show that the intensity IFN-γ T cell responses to KSHV in a Ugandan cohort are very low and infrequent compared to T cell responses to other herpesviruses such as EBV and CMV as well as to influenza virus. While low-intensity IFN-γ T cell responses to KSHV antigens have been reported previously, particularly in patients with KS^2,17,34^, our study (the largest in terms of the number of participants) is the first to report this in apparently healthy HIV-negative individuals in a KSHV endemic region. It may have been expected that in this endemic area, where KSHV reactivation is likely more frequent than in low endemicity regions such as the US, T cell responses to KSHV would have been more prominent. However, the results from the current study are similar to those seen in healthy US participants^20^.

The low level of T cell responses to KSHV differs from the robust T cell responses to other human herpesviruses and requires further investigations. We think, however, that the level of responses is not due to a technical artefact such as the concentration of peptides as we stringently evaluated this possibility in our assay development and validation^20^. Although responses to KSHV are low on average, some individuals had very high responses to certain peptide pools. For example, we observed 3243 SFU per million cells as the maximum response for the K8.1 peptide pool that is very comparable to 3891–SFU per million cells that is the maximum response to the EBV peptide pool. In addition, responses to individual peptides shown in Table 2 are comparable to responses to peptide pools. Furthermore, the number of peptides per KSHV ORF is comparable to the number of peptides in the EBV peptide pool. KSHV has been shown to encode for numerous proteins including host protein homologues that aid host immune evasion^35,36^. For instance, MHC class I, MHC class II, CD1d, CD31, CD54, B7-2, MICA, MICB and IFN-γR1 degradation by KSHV RTA, K5 and K3 proteins had been shown to promote CD4+ T cells, CD8+ T cells and NK cells dysfunction^37^. However, the individuals we studied do not have any form of KSHV-associated disease and are of a wide age range, including older individuals who had probably been infected with KSHV for decades. Therefore, the responses in these individuals, while of low intensity and heterogenous, were sufficient to control KSHV in terms of disease risk. Myoung et al. showed suppression of spontaneous KSHV reactivation in tonsillar B cells by activated tonsillar CD4 T cells in an MHC-independent manner^38^. However, these results have not been replicated and the mechanism of this unspecific KSHV control was not investigated. Future studies investigating these findings further are warranted.

In addition to the T cell responses being low in intensity and frequency, they were very diverse in targeting KSHV antigens across sexes and age groups. Nevertheless, in combination, we have shown that a large number of individuals responded to at least one KSHV antigen (77%). Response to at least one KSHV antigen increases with age and is similar in males and females. This finding is consistent with the early age of infection with KSHV in endemic areas. This implies that the longer someone is infected with the virus, the higher the probability of developing detectable T cells specific for at least one KSHV antigen.

Individuals who responded to one to two KSHV antigens have a lower KSHV viral load compared to those without any detectable KSHV-specific T cell response. Surprisingly, also individuals responding to three or more antigens have a higher KSHV viral load similar to those without any KSHV T cell response. This may imply that T cell response to an individual viral protein such as K8.1 could suppress viral load.

Most studies have failed to show any association between KSHV viral load in PBMC and T cell responses^5,16,30^. This might have been attributed to the small sample sizes of these previous studies. Nevertheless, one study showed increased T cell responses to ORF73 among individuals with detectable KSHV in PBMC^39^, consistent with our observations in this study. Contrary to T cell responses to ORF73, T cell responses to K8.1 are higher in those without KSHV DNA in PBMC, supporting the hypothesis that the presence of KSHV-specific T cell responses inhibits viral replication, hence reducing the risk of detecting the virus in the blood. However, the frequency of this T cell response in KSHV seropositive individuals without detectable viruses is low, implying that there are other mechanisms through which these individuals control the virus.

We also observe reactivity to SIV, a non-human primate virus. This is very surprising because the SIV-CM9 peptide used in the assay is restricted by a common class I allele (Mamu A*01) in macaques. The equivalent epitope in HIV is also restricted by a single HLA allele, yet all the study participants are HIV uninfected. Due to the limitations of sample availability, we could not investigate this observation further, but this is most likely an example of heterologous T cell reactivity.

The strength of this study is the large number of participants compared to most past studies on KSHV T cell responses. In addition, by focusing on HIV uninfected individuals from a KSHV endemic area, as well as including a very wide age range of individuals of both sexes, it allows us to provide a comprehensive evaluation of the T-cell response across the age spectrum in healthy individuals. An additional strength of this study is the use of peptide pools that spanned the KSHV proteome.

In conclusion, IFN-γ T cell responses to KSHV are diverse and when they do occur, they are not as intense as those against EBV, CMV and influenza. However, the responses to particular antigens (for example K8.1) may be more protective from viral reactivation than others (for example ORF73/LANA). These results provide insights into the potential success of immune therapeutic vaccines in preventing or treating KS disease among KSHV-infected individuals at high risk of developing KS.

## Methods

### Ethical approvals

This study is approved by the Uganda Virus Research Institute-Research and Ethics Committee (reference number: GC/127/16/09/566), the Uganda National Council for Science and Technology (reference number: HS2123) and the London School of Hygiene and Tropical Medicine Ethics Committee (reference number: 11881). Written informed consent was obtained from all adults aged 18 years and above. Children below 18 years were consented to the study by their parents or guardian; we also sought, in addition to parental consent, written assent from children aged between 8 and 17 years. Transport refund is provided to all invited study participants following Institution Review Board regulations and approval.

### KSHV ELISPOT assay

Overlapping 15mer peptides are synthesised to span nearly the entire KSHV proteome, and peptides are divided into pools representing each ORF^20^. Using these 83 peptide pools, working concentrations of 5 μg/ml/peptide are prepared in 96-well culture plates using AIM-V media. The MABTECH Human IFN-γ ELISpot kit (Code: 3420-2APT-10) is used for the assay, with a few alterations to the manufacturer’s protocol. The ELISPOT plates with the capture antibody from the kit are washed five times with 200 μl of 1× phosphate-buffered saline (PBS) per well. Afterwards, thawed cells are added to the plates in a volume of 100 μl containing 150,000 cells per well. The plates are covered with the lid and wrapped in aluminium foil and transferred to a 5% CO_2_ 37 °C incubator for a 24 h resting period. To stimulate them, 100 μl per well of the peptide pools are added to the cells. Anti-CD3, a pool of peptides from CMV EBV and Influenza (CEF) plus EBV peptides alone are added as positive controls. Media and a non-human antigen (SIV Gag CM9 peptide) are also added as negative controls. The plates are then incubated at 37 °C for 46–48 h. Following stimulation, cells are washed five times with 200 μl of PBS per well and 100 μl of anti-human IFN-γ IgG conjugated to alkaline phosphatase (Code: 7-B6-ALP) is added at a dilution of 1/200 in PBS + 0.5% FBS. The plates are incubated at room temperature (25 °C) for 2 h. After the incubation, the plates are washed five times with 200 μl of 1× PBS per well and 100 μl of filtered 5-bromo-4-chromo-3-indolyl-phosphate/nitroblue tetrazolium-plus substrate (Code: 3650-10) are added per well. The plates are then incubated at room temperature for 7 min and the reaction is stopped by washing the plate with running tap water. The plates are dried in the dark overnight, subsequently, the spots are counted using an ELISPOT reader (CTL ImmunoSpot Analyzer (Cellular Technology Limited, Shaker Heights, USA)). This protocol has been reported elsewhere^20^. K8.1 peptide pool and an EBV peptide pool were added to the antigen panel at a later time during the ELISpot assay testing hence the first 40 samples missed being tested for these two antigens; 76 samples have a complete panel of antigens including K8.1 and EBV. Peptide sequences for K8.1, EBV and SIV are shown in Supplementary Table 1. Only plates with anti-CD3 responses are included in the analysis. Anti-CD3, CEF and K8.1 SFC/million cells from the 76 samples with the complete antigen panel are shown in Supplementary Fig. 1.

### K8.1 peptide deconvolution

The K8.1 peptide pool (of 44 peptides) is deconvoluted in four study participants who responded to K8.1 peptide pool and had enough PBMC for the assay. Using a grid-type method^20^, 14 smaller overlapping peptide pools of six to seven peptides are generated. IFN-γ responses to the smaller peptide pools are determined using the ELISPOT assay described above. Responding individual peptides identified from the peptide matrix are confirmed by stimulating with these individual responding peptides to run a third IFN-γ ELISPOT assay. The sequences of the peptides identified in the four individuals are 10: VYQDWLGRMNCSYEN, 17: SEYPNVSVSVEDTSA, 22: SGSGEEERPVTSHVT, 29: SGSYSSGEPSRTTRI, 30: SGEPSRTTRIRVSPV, 31: RTTRIRVSPVAENGR, 34: NSGASNRVPFSATTT, 35: NRVPFSATTTTTRGR, 36: SATTTTTRGRDAHYN, 37: TTRGRDAHYNAEIRT, 38: DAHYNAEIRTHLYIL, 41: WAVGLLLGLVLILYL, 43: LILYLCVPRCRRKKP, 44: YLCVPRCRRKKPYIV.

### KSHV ELISA

Plasma samples from these individuals are tested for KSHV IgG antibodies to K8.1 and ORF73 recombinant proteins to confirm seropositivity following the procedure below. DynexImmulon 4 HBX 96-well plates (D17506, Fisher catalogue number: NC9939836) are coated with 100 μl of K8.1 and ORF73 recombinant proteins at a dilution of 1:5000 for each protein. K8.1 protein is diluted in 0.05 M carbonate/bicarbonate buffer, pH 10 and ORF73 in 1× PBS. The plates are sealed using Nunc plate sealers and incubated in a fridge (4 °C) overnight. After the overnight incubation, plates are washed three times with 350 μl of wash solution (1× PBS, 0.05% Tween-20) per well, using an automated plate washer (BioTek ELx405). They are then inverted and tapped dry on paper towels. A volume of 270 μl assay buffer (2.5% bovine serum albumin-BSA (Sigma Chemical, catalogue number: A-7284) plus 2.5% normal donor goat serum (Equitech-Bio catalogue number SG-0500) and 0.005% Tween-20 as well as 0.005% Triton X-100 in 1× PBS) is added to each plate as a blocking agent, sealed with a plate sealer, and incubated for 3 h at 37 °C and stored at −80 °C. To run the assay, plates are thawed and washed three times with 350 μl of wash solution prior to adding samples. Plasma samples and controls diluted 1:20 for K8.1 and 1:100 for ORF73 in assay buffer are added to each plate in a volume of 100 μl. Plates are sealed and incubated in a 37 °C incubator for 90 min. Each plate contains 88 samples, negative and positive controls (each in triplicate) and a blank (assay buffer) in duplicate. After the incubation, plates are washed five times with wash buffer, tapped dry on paper towels, and 100 μl of goat anti-human IgG-Alkaline phosphatase labelled conjugate (KPL catalogue number 4751-1002) at a dilution of 1:5000 in assay buffer was added per well. Plates are then sealed and incubated at 37 °C for 30 min. Following the 30 min incubation, plates are washed five times, tapped dry on a paper towel, and 100 μl of 1-step p-nitrophenyl phosphate substrate solution is added per well. They are then developed in the dark at room temperature for 30 min for ORF73 and 25 min for K8.1. Plates are then immediately read using a microtiter plate reader (BioTek ELx808) at a wavelength of 405 nm. Optical densities (ODs) are obtained. The ODs of the blank wells are used for background subtraction for each sample and control. The positive and negative controls are used for quality control, to determine if a plate had passed. The negative controls are also used to calculate a cut-off value for each plate. The cut-off value for each plate is the average background-subtracted OD of the three negative control triplicates plus a constant value of 0.75 (for K8.1) or 0.35 (for ORF73).

### KSHV PCR

KSHV viral load is quantified from PBMC KSHV seropositive study participants. After PBMC isolation using ficoll density gradient method, two million cells are removed, spun at 13,000 rcf for 10 min to form cell pellets. The supernatants are poured off and the cell pellets are stored at −80 °C. The PBMC pellets are retrieved from the −80 °C freezer, thawed and genomic DNA extracted using a QIAamp blood kit (Qiagen, Valencia, CA), following the manufacturer’s instructions. KSHV viral load is quantified using real-time PCR. KSHV DNA is detected using primers (K6 forward primer K6-10F 5′-CGCCTAATAGCTGCTGCTACGG-3′, K6 reverse primer K6-10R 5′-TGCATCAGCTGCCTAACCCAG-3′) and a probe (K6 probe p-K6-10 5′-R-CACCCACCGCCCGTCCAAATTC-Q-3′) previously reported to be specific to the K6 gene region^40^. In addition, the number of cellular equivalents are determined using a quantitative assay specific to human endogenous retrovirus 3 (ERV-3), which is present in two copies per genomic cell, using these primers (ERV-3 Forward primer PHP10-F 5′-CATGGGAAGCAAGGGAACTAATG′ ERV-3 Reverse primer PHP10-R 5′-CCCAGCGAGCAATACAGAATTT-3′) and a probe (ERV-3 Probe PHP-P505 5′-R-TCTTCCCTCGAACCTGCACCATCAAGTCA-Q-3′). To quantify both ERV-3 and KSHV DNA, seven two-fold serial dilutions of K6 and ERV-3 are made from clone stocks (starting with 1 × 10^6^ dilutions to 1 × 10^0^) to form a standard curve on every plate. ERV-3 was cloned into Bluescript II KS vector (Stratagene, La Jolla, CA, USA) KSHV K6 cloned using PCR Topo II vector, Topo TA Cloning kit, Invitrogen, K 4600-40. Primers (500 μl each), probes (500 μl), nuclease-free water (5.5 ml) and universal master mix (Applied Biosystems, Foster City CA) (12.5 ml) are mixed together to form the working solution master mix. Thereafter 10 μl of the standards, sample and controls each, are added to a 96-well PCR plate (ThermoFisher catalogue number 7306737) per well, in triplicate, and 40 μl of the working solution master mix added to each well. This was followed by plate sealing and DNA amplification using an ABI ViiA7 machine. All samples are tested in triplicate for both assays and the estimated copy number for each individual reaction is averaged. The KSHV viral load is determined in PBMC DNA by calculating the viral DNA copies per million cells. Any sample that is quantifiable by the ERV-3 assay but not positive in all three reactions in the KSHV K6 assay is designated as positive but not quantifiable. The lower limit of detection for the ERV-3 assay has been previously determined as 10 copies per million cells while the KSHV K6 assay has a lower limit of sensitivity of 1 copy per million cells. This procedure has been reported elsewhere^41–43^. In order to prevent contamination, qPCR reagents, sample processing, DNA extractions, and qPCR setup are conducted in dedicated laboratory areas. Internal no template controls (NTC) were included on each plate, in triplicate, to assess cross-contamination. Assay quality controls including NTC, assay controls and sample triplicate % coefficient of variation are used to validate data. Importantly, the individual dilution parameters of the assay standard curve are assessed after each run to ensure performance within expected ranges. Any assay that has failures of assay controls or standard curves is repeated.

### Statistics and reproducibility

#### Study population and study design

The General Population Cohort (GPC) is a community-based cohort of about 22,000 people living in 25 adjacent villages^44,45^, in rural southwestern Uganda. Previously, we have documented a high seroprevalence (>90%) of KSHV in the GPC^46,47^. For this study, we recruited individuals from the GPC who were KSHV seropositive, but HIV seronegative in 2017, to investigate determinants of KSHV viral detection. Only one individual was recruited per household^26^. Blood samples were processed to isolate PBMC (stored in liquid nitrogen for later immunological assays) and plasma (stored at −80 °C for later use). Cell pellets containing two million PBMC were stored at −80 °C for later DNA extraction. Details of the main study design have been reported previously^26^. KSHV-specific T cell IFN-γ responses are determined in 116 participants aged 6–87 years. No statistical method is used to predetermine sample size. Purposive sampling is used to ensure that both male and female participants with and without detectable viral DNA in PBMC from each age group are selected. Simple randomisation is used to select individuals in each age group. Investigators are blinded to allocation during experiments and recruitment. Details of the characteristics of the participants (including the sex and age of participants) selected are shown in Table 1.

Graphical representation is done using GraphPad Prism 8.0.1 and STATA version 13 (StataCorp, College Station, Texas USA). SFC per million PBMC are calculated from the number of spots counted by the ELISpot reader and the number of PBMC plated using Microsoft Office Excel (version 15.23). A cut-off of 40 SFC is applied. Therefore, individuals with SFC below 40 are considered non-responders. *χ*^2^ test, Kruskal–Wallis test, Mann–Whitney (Wilcoxon rank-sum) test are used for statistical analysis in STATA version 13 (StataCorp, College Station, Texas USA). No data were excluded from the analysis.

### Reporting summary

Further information on research design is available in the Nature Research Reporting Summary linked to this article.

## Supplementary information

Supplementary Information

Description of Additional Supplementary Files

Supplementary Data 1

Supplementary Data 2

Reporting Summary

## Data Availability

The data shown in all figures and tables generated in this study are provided in the Supplementary information/Source Data file. Source Data are provided with this paper.

## Source data

Source Data
