# Supplementary material for: Kaposi’s sarcoma-associated herpesvirus T cell responses in HIV seronegative individuals from rural Uganda
Source: Nat Commun. 2021 Dec 16;12:7323. doi: 10.1038/s41467-021-27623-8 (PMC8677732; doi:10.1038/s41467-021-27623-8)
Supplement: Supplementary file 1 — Supplementary Information [file 41467_2021_27623_MOESM1_ESM.pdf]

Kaposi's sarcoma-associated herpesvirus T cell responses in HIV seronegative individuals from rural Uganda

Supplementary Information

Angela Nalwoga[1,2] , Romin Roshan[3], Kyle Moore[3], Vickie Marshall[3], Wendell Miley[3], Nazzarena Labo[3], Marjorie Nakibuule[1], Stephen Cose[1,4], Rosemary Rochford[2], Robert Newton[5] and Denise Whitby[3]

1. MRC/UVRI and LSHTM Uganda Research Unit, Entebbe; Uganda
2. Department of Immunology and Microbiology, University of Colorado, Anschutz Medical Campus, Aurora, Colorado; United States of America
3. Viral Oncology Section, AIDS and Cancer Virus Program, Leidos Biomedical Research, Inc., Frederick National Laboratory for Cancer Research, Frederick, MD; United States of America
4. London School of Hygiene & Tropical Medicine, London; United Kingdom
5. University of York, York; United Kingdom

Supplementary Figure 1: Anti-CD3, CEF and EBV IFN-γ responses of 76 Ugandans aged 18-50 years.

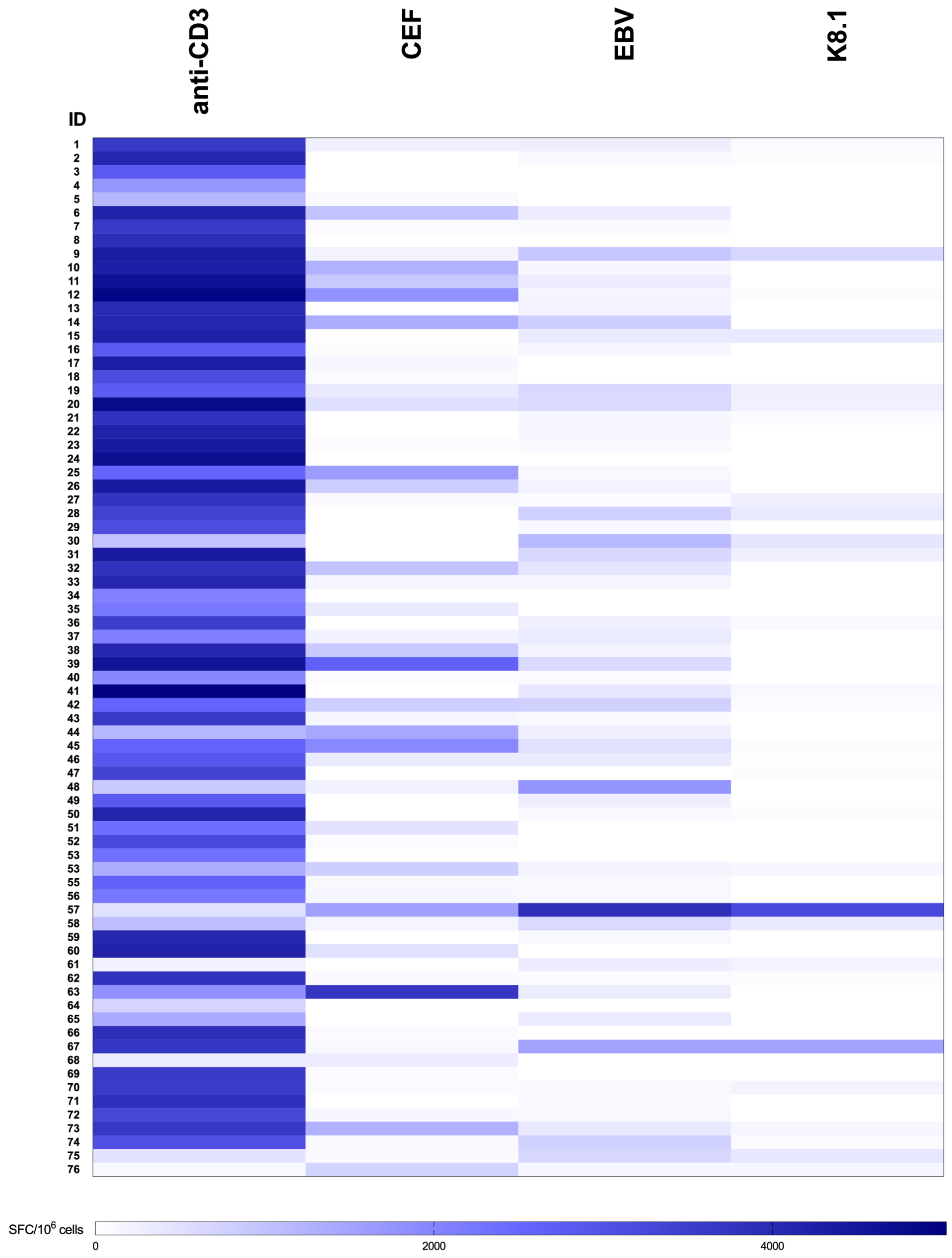

Ex-vivo ELISpot assay was used to determine IFN-γ responses. Spot forming cells (SFCs) per million PBMCs were recorded for each reaction. The intensity of the purple colour correlates with the number of SFCs per million PBMCs. EBV: Epstein-Barr virus, CEF: CMV+EBV+flu cocktail. ID: study participant identification. Heatmap drawn in GraphPad Prism version 8.0.1. Source data are provided as a Source Data file.

Supplementary Figure 2: Relationship between number of reactive KSHV peptide pools and age of the study participant.

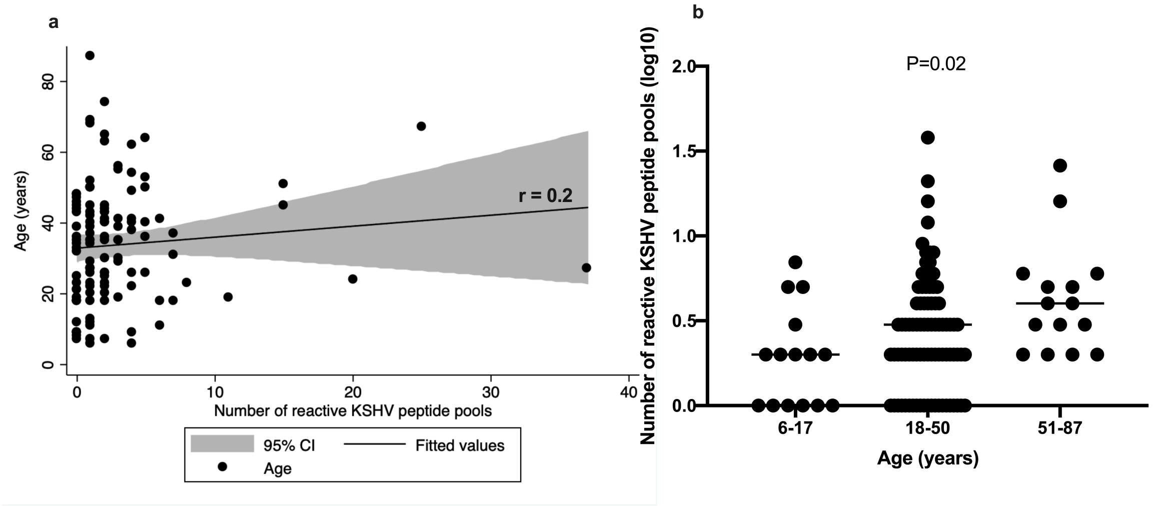

A-Correlation between age and number of reactive peptide pools (A). B-Number of reactive peptide pools by age group. Graphs were drawn using STATA and GraphPad Prism version 8.0.1 while correlation coefficient obtained in STATA version 13.0. N=116 study participants. Fig B: Dot plot indicate median. P-value obtained from a two sided Kruskal Wallis test to avoid multiple comparisons. Source data are provided as a Source Data file.

Supplementary Figure 3: Correlation between IFN-γ responses to K8.1 and IgG antibody responses to K8.1. IFN-γ responses were measured in PBMC using ex-vivo ELISpot assay.

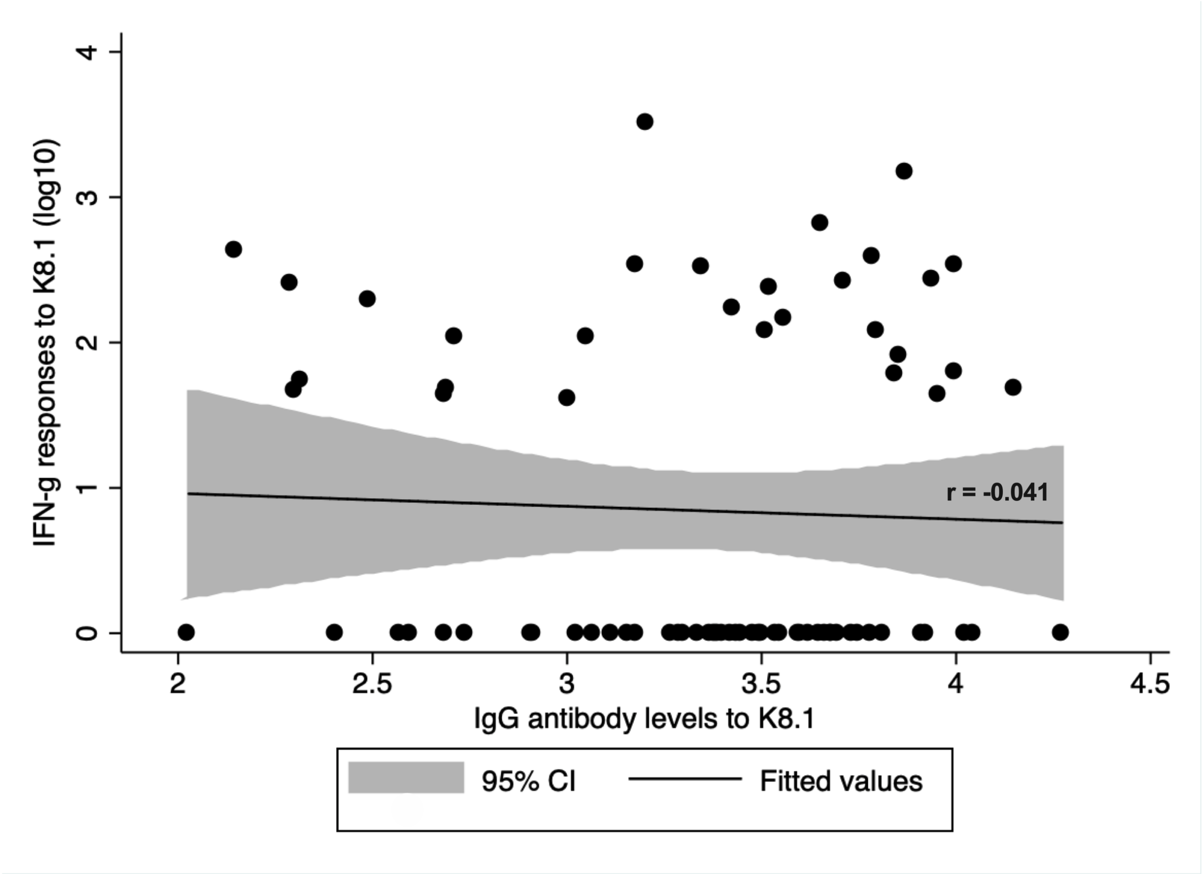

IgG responses were measured using a multiplex bead assay. Correlation coefficient obtained in STATA version 13.0. N=76 study participants. Source data are provided as a Source Data file.

Supplementary Table 1:K8.1, EBV and SIV peptide pool sequencies.

protein/virus	Peptide pool sequence/details
K8.1	MSSTQIRTEIPVALL, IRTEIPVALLILCLC, PVALLILCLCLVACH, ILCLCLVACHANCPT, LVACHANCPTYRSHL, ANCPTYRSHLGFWQE, YRSHLGFWQEGWSGQ, GFWQEGWSGQVYQDW, GWSGQVYQDWLGRMN, VYQDWLGRMNCSYEN, LGRMNCSYENMTALE, CSYENMTALEAVSLN, MTALEAVSLNGTRLA, AVSLNGTRLAAGSPS, GTRLAAGSPSSEYPN, AGSPSSEYPNVSVSV, SEYPNVSVSVEDTSA, VSVSVEDTSASGSGE, EDTSASGSGEDAIDE, SGSGEDAIDESGSGE, DAIDESGSGEEERPV, SGSGEEERPVTSHVT, EERPVTSHVTFMTQS, TSHVTFMTQSVQATT, FMTQSVQATTELTDA, VQATTELTDALISAF, ELTDALISAFSGSYS, LISAFSGSYSSGEPS, SGSYSSGEPSRTTRI, SGEPSRTTRIRVSPV, RTTRIRVSPVAENGR, RVSPVAENGRNSGAS, AENGRNSGASNRVPF, NSGASNRVPFSATTT, NRVPFSATTTTTRGR, SATTTTTRGRDAHYN, TTRGRDAHYNAEIRT, DAHYNAEIRTHLYIL, AEIRTHLYILWAVGL, HLYILWAVGLLLGLV, WAVGLLLGLVLILYL, LLGLVLILYLCVPRC, LILYLCVPRCRRKKP and YLCVPRCRRKKPYIV
Epstein Barr virus (EBV)	CLGGLLTMV, GLCTLVAML,FLDKGTYTL, FLYALALLL, YLLEMLWRL, RVRAYTYSK, RLRAEAQVK, ATIGTAMYK, AVFDRKSDAK, IVTDFSVIK, SSCSSCPLSK, DYCNVLNKEF, PYLFWLAAI, TYGPVFMCL, TYGPVFMSL, RPPIFIRRL, QPRAPIRPI, FLRGRAYGL, RAKFKQLL, RRIYDLIEL, YPLHEQHGM, EPLPQGQLTAY, HPVGEADYFEY, IEDPPFNSL, EENLLDFVRF, QAKWRLQTL, RPQGGSRPEFVKL, DSELEIKRYKNR, ARYAYYLQF, AENAGNDAC, SVRDRLARL, LLDFVRFMGV, TYSAGIVQI, VFSDGRVAC, AYSSWMYSY, VEITPYKPTW, VSFIEFVGW
Simian Immunodeficiency virus (SIV) Gag CM9 peptide	gag protein (181-189) SIV. Gene: Ac-CTPYDINQM-amide (9mer). MW: 1125 mg:1.5 Cat#: BP12-131.
