# Supplementary material for: Kaposi’s sarcoma-associated herpesvirus T cell responses in HIV seronegative individuals from rural Uganda
Source: Nat Commun. 2021 Dec 16;12:7323. doi: 10.1038/s41467-021-27623-8 (PMC8677732; doi:10.1038/s41467-021-27623-8)
Supplement: Supplementary file 2 — Description of Additional Supplementary Files [file 41467_2021_27623_MOESM2_ESM.pdf]

Description of Additional Supplementary Files

Supplementary Data 1: KSHV specific IFN-g responses (SFC per million cells) n=40

Supplementary Data 1: Ex-vivo ELISpot assay was used to determine IFN-γ responses to KSHV overlapping peptide pools. Spot forming cells (SFCs) per million PBMCs were recorded for each reaction. SIV: Simian immunodeficiency virus, CEF: CMV+EBV+flu cocktail

Supplementary Data 2: KSHV specific IFN-g responses (SFC per million cells) n=76

Supplementary Data 2: Ex-vivo ELISpot assay was used to determine IFN-γ responses to KSHV overlapping peptide pools. Spot forming cells (SFCs) per million PBMCs were recorded for each reaction. SIV: Simian immunodeficiency virus EBV: Epstein-Barr virus, CEF: CMV+EBV+flu cocktail.
