# Supplementary material for: Kaposi’s sarcoma-associated herpesvirus T cell responses in HIV seronegative individuals from rural Uganda
Source: Nat Commun. 2021 Dec 16;12:7323. doi: 10.1038/s41467-021-27623-8 (PMC8677732; doi:10.1038/s41467-021-27623-8)
Supplement: Supplementary file 5 — Reporting Summary [file 41467_2021_27623_MOESM5_ESM.pdf]

nature portfolio

Corresponding author(s): Nalwoga A., Whitby D.

Last updated by author(s): Nov 2, 2021

Reporting Summary

Nature Portfolio wishes to improve the reproducibility of the work that we publish. This form provides structure for consistency and transparency in reporting. For further information on Nature Portfolio policies, see our Editorial Policies and the Editorial Policy Checklist.

Statistics

For all statistical analyses, confirm that the following items are present in the figure legend, table legend, main text, or Methods section.

n/a	Confirmed	
☐	☒	The exact sample size (n) for each experimental group/condition, given as a discrete number and unit of measurement
☐	☒	A statement on whether measurements were taken from distinct samples or whether the same sample was measured repeatedly
☐	☒	The statistical test(s) used AND whether they are one- or two-sided<br>*Only common tests should be described solely by name; describe more complex techniques in the Methods section.*
☐	☒	A description of all covariates tested
☐	☒	A description of any assumptions or corrections, such as tests of normality and adjustment for multiple comparisons
☐	☒	A full description of the statistical parameters including central tendency (e.g. means) or other basic estimates (e.g. regression coefficient) AND variation (e.g. standard deviation) or associated estimates of uncertainty (e.g. confidence intervals)
☐	☒	For null hypothesis testing, the test statistic (e.g. F, t, r) with confidence intervals, effect sizes, degrees of freedom and P value noted<br>*Give P values as exact values whenever suitable.*
☒	☐	For Bayesian analysis, information on the choice of priors and Markov chain Monte Carlo settings
☒	☐	For hierarchical and complex designs, identification of the appropriate level for tests and full reporting of outcomes
☒	☐	Estimates of effect sizes (e.g. Cohen's d, Pearson's r), indicating how they were calculated

Our web collection on statistics for biologists contains articles on many of the points above.

Software and code

Policy information about availability of computer code

Data collection	Data was collected using Microsoft Access
Data analysis	softwares used to carry out data analysis include GraphPad Prism 8.0.1, STATA version 13 (StataCorp, College Station, Texas USA), Microsoft Office Excel (version 15.23).

For manuscripts utilizing custom algorithms or software that are central to the research but not yet described in published literature, software must be made available to editors and reviewers. We strongly encourage code deposition in a community repository (e.g. GitHub). See the Nature Portfolio guidelines for submitting code & software for further information.

Data

Policy information about availability of data

All manuscripts must include a data availability statement. This statement should provide the following information, where applicable:

- Accession codes, unique identifiers, or web links for publicly available datasets
- A description of any restrictions on data availability
- For clinical datasets or third party data, please ensure that the statement adheres to our policy

Provide your data availability statement here

Field-specific reporting

Please select the one below that is the best fit for your research. If you are not sure, read the appropriate sections before making your selection.

☒ Life sciences ☐ Behavioural & social sciences ☐ Ecological, evolutionary & environmental sciences

For a reference copy of the document with all sections, see nature.com/documents/nr-reporting-summary-flat.pdf

Life sciences study design

All studies must disclose on these points even when the disclosure is negative.

Sample size	116 individuals were analyzed, based on availability of resources. no sample size calculations were carried out prior to the selection. This study has the largest sample size for this kind of experiments. Future studies will base on these results to make sample size calculations.
Data exclusions	No data available was excluded from the analysis
Replication	Every ELISPOT plate had three sets of control replicates which had to pass a uniform criteria as shown in the methods of the manuscript for the results on that plate to be considered. All assays used were validated by the Frederick National Laboratory for Cancer Research, USA.
Randomization	To avoid self selection bias or any other biases, simple randomization was used to select participants in each age group ensuring that every person in each age group had an equal chance of being selected
Blinding	Investigators were blinded of the study groups while collecting data and carrying out laboratory experiments.

Reporting for specific materials, systems and methods

We require information from authors about some types of materials, experimental systems and methods used in many studies. Here, indicate whether each material, system or method listed is relevant to your study. If you are not sure if a list item applies to your research, read the appropriate section before selecting a response.

Materials & experimental systems

n/a	Involved in the study
☒	☐ Antibodies
☒	☐ Eukaryotic cell lines
☒	☐ Palaeontology and archaeology
☒	☐ Animals and other organisms
☐	☒ Human research participants
☒	☐ Clinical data
☒	☐ Dual use research of concern

Methods

n/a	Involved in the study
☒	☐ ChIP-seq
☒	☐ Flow cytometry
☒	☐ MRI-based neuroimaging

Human research participants

Policy information about studies involving human research participants

Population characteristics	All study participants were from the same geographical area, their age ranged from 6 to 87 years with a mean age of 34 years. 51% of these were males while the rest were female.
Recruitment	KSHV seropositive individuals were selected from the database using previous population based results. The selection was weighted to include equal numbers in each age group. To avoid self selection bias or any other biases, simple randomization was used to select participants in each age group ensuring that every person in each age group had an equal chance of being selected. All consented, eligible individuals were included in the main study.
Ethics oversight	The Uganda Virus Research Institute Research and Ethics Committee, The Uganda National Council for Science and Technology and LSHTM ethics Committee

Note that full information on the approval of the study protocol must also be provided in the manuscript.
